# Rheumatism and Gout

**Published:** 1904-04-02

**Authors:** 


*(To be continued.)*

### Rheumatism and Gout.

*(Continued from Vol. XXXV., page 426.)*

**Sciatica** (*continued*).—J. Michell Clarke[10] recommends for obstinate cases immobilisation of the affected limb by a long splint from the axilla to the foot. Of the 12 last consecutive cases thus treated, 10 were cured, one would not submit to the splint and one had a relapse after some weeks, but was cured by a second course of treatment. The thigh is first carefully bandaged by a firm flannel bandage. The splint is then fixed to the trunk and leg, the knee being in slight flexion, and the heel carefully supported. After the first few days the bandages are taken off daily, and reapplied at once with as little disturbance as possible. At the end of the first week gentle passive movement of the joints is made when the bandages are thus changed. When the pain has been lost for some days, usually in two or three weeks, the splint is left off by day and applied by night, and finally left off altogether. A week later the patient may be allowed to get up, but not to sit down. The average duration of the illness in his cases before treatment was 23.6 weeks, and the average time till the splint was given up was 25 days. The first day or two is very trying, and morphia may be needed for one or two nights, and much persuasion may be necessary to induce patients to submit, but after a few days great relief is felt. Flatulent dyspepsia must be combated, and the bed should be firm and flat. William Bruce, in a paper before the Medical Graduates' College, argues that sciatica is due to an affection of the hip joint, and not of the sciatic nerve, and that it goes on to rheumatoid arthritis of the joint, and sometimes to ankylosis. In 55 per cent. of his cases he found tenderness over the capsule of the joint, and in 52 per cent. gout or rheumatism in other joints.

**Chronic Rheumatism.**—Ralph Stockman[11] takes the view that this is a definite disorder quite distinct from acute rheumatism, rheumatoid, or septic arthritis. It is due to inflammatory indurations of the fibrous ligaments, fasciæ, and sheaths of muscles and nerves, together with exudation, which may become organised and form fibrous swellings. These swellings vary in size from a small shot upwards, or may form a thick cord; they are liable to become painful under the influence of cold, dyspepsia, fatigue, or a strain. Thus there may be continual aching and a sense of fatigue. The original causes of these indurations may be acute rheumatism, influenza, or exposure to cold, and when once formed, painful or irritable conditions are easily set up. For these temporary exacerbations there are numerous remedies; for a permanent cure the indurations must be dispersed by (1) massage, (2) exercises, (3) faradism, (4) or injection of chromic acid. Massage is the chief means. The nodules must be searched for and skilfully rubbed daily for 10 or 15 minutes for weeks. Deeply seated and well defined nodules may be injected with 10 minims of the chromic acid solution of one per cent. strength.

**Rheumatoid Arthritis.**—Macalister[12] remarks on the origin of some cases in septic infections of the mucous membranes, and of others from thyroid changes. The poison seems to be like that of ergot, and is not easily eliminated. Thyroid treatment in a few cases does good. *Pulmonary osteoarthropathy*, according to Symes Thompson[13] shows symmetrical bony enlargements with intermittent effusion in the joints, and clubbing of the fingers, and usually follows a suppurative process in the chest. Toxins are volatile or non-volatile. The latter are eliminated by the liver, and the volatile by the lungs, and if the work of the lungs is much interfered with the retained toxins may produce those bone and joint changes. Still it is difficult to understand the cases which arise in congenital heart disease. S. W. Curl reported an instance of this rare disease last year from the Ventnor Consumption Hospital, and could only find 25 others during 10 years in English and American Journals. Janeway[14] describes two new cases, and remarks that in some instances no previous lung or heart affection has existed. The ordinary clubbed fingers of heart disease are closely related to this disease, in the latter indeed, there is no bony enlargement of the terminal phalanges any more than in ordinary clubbed fingers. If the selective action of the toxins absorbed from suppuration is the explanation of many of the cases, how do those cases begin which are not secondary to lung and other diseases? Out of the 93 cases which Janeway has collected, there are nine without previous disease elsewhere.

Arthritis, with ankylosis attacking the spinal column, is discussed by Ruhräh[15] under the name of *Spondylitis deformans*. There is a more or less complete fusion of the vertebræ, and, according to French writers, of the proximal articulations, but not of the small joints. The ribs may become fixed, and there are large abnormal deposits of new bone around and between the vertebræ. The pain may lead to its being mistaken for Pott's disease, but generally decreases as the disease progresses. Ruhräh would distinguish it from rheumatoid arthritis, chronic rheumatism, and pachymeningitis, and notices that instances of the disease have even been found in Egyptian mummies. Nutritious food, iron, arsenic, and cod-liver oil are recommended, with continued rest in bed, and plaster jackets to support the spine till the disease has come to a quiescent stage.

**Purpura.**—E. Lenoble[16] finds a definite type which has the following marks: The blood does not freely separate into serum and clot; there are normoblasts and myelocytes present; the platelets are always diminished in number, increased in size, and are powerless to undergo spontaneous change; hæmoglobin and the red corpuscles are equally diminished, and poikilo-cytosis is present, while white cells are moderately increased; the lymphocytes increasing with each hæmorrhage for a time. These cases are marked also by profuse hæmorrhages, and on the whole the changes are those seen in mild cases of myeloid leukæmia. Herringham[17] finds that when purpura complicates cancer, it occurs almost always in sarcoma, and rarely in carcinoma, except for a few petechiæ in the final anæmia. Now the former type is disseminated by the blood, and the latter by the lymph, and the hæmorrhages are probably due to the action of sarcoma cells on the blood-vessels, similar to that of the organisms and toxins in septicæmia. So, too, are produced the hæmorrhages from the lungs and kidneys in general miliary tuberculosis. H. G. Macadam[18] reports a severe case of hæmorrhagic purpura in a child which he treated successfully with large doses of adrenalin. Kinsay had a similar experience, and Jacobson another, in which the hæmorrhages into the substance of the appendix led to laparotomy for supposed appendicitis.

**Hæmophilia.**—Wachenheim[19] thinks the disease is a toxæmia, which has gone on to auto-immunisation. Thus in obstructive jaundice there is a similar, but temporary, condition in which agglutinins are formed, showing the presence of a toxin. Hæmorrhages again may occur in any of the infectious diseases for they are all toxæmias. H. B. Pearson[20] reports a case of hæmaturia, lasting some three years, with other hæmorrhages at times, occurring in a hæmophiliac patient. The family was peculiar in that the disease, instead of being transmitted to males alone, appeared first in a woman, and affected her male and female descendants equally. P. S. Blaker[21] also mentions the case of a female child, who inherited the disease from the father's mother, while the males of the family escaped.

[10] Lancet, October 17. [11] Brit. Med. Jour., February 27. [12] Ibid., November 21. [13] Lancet, January 16. [14] Amer. Jour. Med. Sc., October. [15] Ibid., November. [16] Birm. Med. Rev., October. [17] Birm. Med. Rev., Oct. [18] Med. Rec., Aug. [19] Med. Rec., Jan. 23. [20] Lancet, Jan. 4. [21] Lancet, Jan. 23.

